# Quantifying the direct radiative effect of absorbing aerosols for numerical weather prediction: a case study

**DOI:** 10.5194/acp-19-205-2019

**Published:** 2019-01-01

**Authors:** Mayra I. Oyola, James R. Campbell, Peng Xian, Anthony Bucholtz, Richard A. Ferrare, Sharon P. Burton, Olga Kalashnikova, Benjamin C. Ruston, Simone Lolli

**Affiliations:** 1 American Society for Engineering Education, Monterey, CA 93943, USA; 2 US Naval Research Laboratory, Monterey, CA 93943, USA; 3 NASA Langley Research Center, Langley, VA 23681, USA; 4 Jet Propulsion Laboratory, Pasadena, CA 91109, USA; 5 CNR-IMAA, Istituto di Metodologie per l’Analisi Ambientale, Tito Scalo (PZ), Italy

## Abstract

We conceptualize aerosol radiative transfer processes arising from the hypothetical coupling of a global aerosol transport model and a global numerical weather prediction model by applying the US Naval Research Laboratory Navy Aerosol Analysis and Prediction System (NAAPS) and the Navy Global Environmental Model (NAVGEM) meteorological and surface reflectance fields. A unique experimental design during the 2013 NASA Studies of Emissions and Atmospheric Composition, Clouds and Climate Coupling by Regional Surveys (SEAC^4^RS) field mission allowed for collocated airborne sampling by the high spectral resolution Lidar (HSRL), the Airborne Multi-angle SpectroPolarimetric Imager (AirMSPI), up/down shortwave (SW) and infrared (IR) broadband radiometers, as well as NASA A-Train support from the Moderate Resolution Imaging Spectroradiometer (MODIS), to attempt direct aerosol forcing closure. The results demonstrate the sensitivity of modeled fields to aerosol radiative fluxes and heating rates, specifically in the SW, as induced in this event from transported smoke and regional urban aerosols. Limitations are identified with respect to aerosol attribution, vertical distribution, and the choice of optical and surface polarimetric properties, which are discussed within the context of their influence on numerical weather prediction output that is particularly important as the community propels forward towards inline aerosol modeling within global forecast systems.

## 1. Introduction

Over the last two decades much progress has been achieved in terms of characterizing aerosol properties, identifying their spatiotemporal extent, and determining their role in the planetary radiative balance (Ramanathan et al., 2001). As a result of that endeavor, the scientific community has been able to recognize that aerosols have a “direct effect” on climate by modifying the planet’s radiative budget and redistributing heat in the atmosphere, and an “indirect effect” by modifying cloud development, precipitation, and optical properties (IPCC, 2014). Additionally, it is implicit that these effects are reliant on aerosol altitude, and on the reflectance (albedo) of the underlying surface (Lyapustin et al., 2011; Bauer and Menon, 2012; Xu et al., 2017).

Nevertheless, significant uncertainty still remains when it comes to understanding the atmosphere’s response to different aerosol physical properties, particularly on day-to-day scales that impact weather (Mulcahy et al., 2014; Toll et al., 2016; Zhang et al., 2016). Aerosols are now regular components of numerical weather prediction models (NWP), and it has been shown through model sensitivity studies that aerosol radiative coupling effects are not trivial regarding their influence on resolved weather processes (Carmona et al., 2008; Milton et al., 2008; Mulcahy et al., 2014; Toll et al., 2016). For example, increased aerosol scattering and absorption of incoming shortwave (SW) and outgoing longwave radiation (OLR) fields modify the atmospheric heating profile and can affect both large-scale and regional circulation patterns (Haywood et al., 2005; Mulcahy et al., 2010). Furthermore, the omission of the scattering and absorption properties, in particular for mineral dust and biomass burning, was identified in case study analysis as the principal cause of significant biases (in the order of 50–56 Wm^−2^, over dust source regions) in both model OLR at the top-of-atmosphere (TOA) (Haywood et al., 2005) and surface SW radiation fields (Milton et al., 2008).

Until recently, the representation of aerosols in global NWP systems at most weather offices was based on a simple aerosol climatology or monthly averages of aerosol concentrations and optical properties (e.g., Tegen et al., 1997), which omit the daily variability of these constituents and thus do not account for changes in concentration, size, and vertical distribution. While models show fundamental improvement when considering aerosols (such as the reflected SW radiative bias at the TOA), temperature biases in the lower troposphere of approximately 0.5 K day^−1^ were documented by Mulcahy et al. (2010) due to the aerosol climatology being too absorbing. These biases, in turn, translate into spatiotemporal discrepancies in precipitation and temperature forecasts (Carmona et al., 2008; Milton et al., 2008).

Examples of significant improvement found in NWP skill when considering aerosols include forecasts of the African Easterly Jet at the European Centre for Medium-Range Forecast (ECMWF) and reduction of temperature and precipitation seasonal mean-biases (e.g., Tompkins et al., 2005; Rodwell and Jung, 2008). In the case of real-time or near real-time prognostic aerosols, Mulcahy et al. (2014) demonstrated an overall improvement in the NWP radiative budget fields by means of an improved representation of the direct radiative forcing, while Toll et al. (2015, 2016) demonstrated improvement in forecast of near-surface fields over extreme aerosol events, such as the 2010 fires that occurred in Russia.

Despite the potential benefits of proper aerosol characterization in NWP systems, aerosols physics have to date not been fully coupled with the operational weather modeling components for a number of reasons, including the following: (a) inaccurate model initialization due to limited knowledge of the aerosol spatiotemporal distribution, particularly in the vertical (Alpert et al., 2002; Zhang et al., 2011, 2014); (b) the physical/chemical effects of aerosols on the atmospheric energy balance, and in particular their various interactions with clouds, are not well constrained (Ramanathan et al., 2001; Jacobson and Kaufman, 2006); and (c) added demand on computational requirements. However, advances in data assimilation schemes for NWP applications, combined with the development of accurate, standalone, 3-D aerosol models mean that some of these limitations can now be circumvented. The capabilities for an “inline” aerosol model, (one that runs in parallel and coupled with the NWP model) have been developed and implemented at a few centers, including the European Center for Medium-Range Weather Forecast (ECMWF – Mulcahy et al., 2014) and NASA GMAO (Randles et al., 2017), and the model is in the process of being implemented at the US Naval Research Laboratory, Marine Meteorology Division. However, such a venture is not trivial and the implications need to be characterized.

Here, we combine operational prognostic aerosol model profiles and a global weather model analysis into a four-stream radiative transfer model, with the express goal of evaluating how well the aerosol model achieves column radiative closure relative to its depiction of the vertical mass concentration profile. We evaluate data generated during the 2013 Studies of Emissions and Atmospheric Composition, Clouds and Climate Coupling by Regional Surveys (SEAC^4^RS), combined with coincident satellite-derived surface reflectance data. The SEAC^4^RS datasets represent a unique opportunity to attempt this model evaluation experiment, given the instrumentation flown aboard two collocated aircraft that simultaneously measured the vertical aerosol profile at high resolution and airborne up/down broadband solar and infrared radiative fluxes. Thus, we attempt radiative closure with the in situ instrumentation and use it to evaluate model skill in depicting aerosol radiative properties. More specifically, and within the observational constraints of the limited dataset available from which to attempt this study, we aim to understand the magnitude of aerosol heating rates, (particularly those associated with transported smoke and urban aerosols), evaluate surface sensitivity to smoke properties, and assess the radiative impact of smoke layers and their potential influence on NWP outputs.

## 2. Data and methods

SEAC^4^RS was conducted in August and September 2013, and was focused primarily on the southeastern US; the objective of the field mission was understanding how summer storms and air pollution from wildfires, cities, and other sources impact climate (Toon et al., 2016). As such, a very comprehensive suite of observations from satellites, aircraft, and ground sites were combined, providing the unique opportunity to characterize the radiative effects of aerosols on the basis of their spectral optical properties. In this study, we apply these measurements for process evaluation relative to prognostic aerosol and NWP model fields. In the following we describe the tools employed.

### 2.1 High spectral resolution lidar (HSRL)

During SEAC^4^RS, aerosol vertical information was collected by the NASA Langley Research Center (LaRC) airborne ozone differential absorption lidar and aerosol/cloud high spectral resolution lidar-2 (DIAL/HSRL) (Hair et al., 2008), which was flown on the NASA DC-8 aircraft. The NASA Langley airborne HSRL instrument technique has been described elsewhere (e.g., Hair et al., 2008; Burton et al., 2012, 2013). In short, the HSRL makes direct measurements of aerosol intensive properties, such as the aerosol backscatter coefficient (*β*) and the depolarization ratio (*δ*) at 355, 532, and 1064nm wavelengths, and the aerosol extinction coefficient (*α*) at a wavelength of 532 nm. Data are sampled at 2 Hz and 15 m vertical resolution, and are then horizontally averaged for 10s (*β* and *δ*) and 60s (*α*). However, the nominal resolution for the backscatter and depolarization (extinction) is 30 m (300 m) in the vertical and 2 km (12 km) horizontally.

Of note for this experiment, aerosol characterization using the HSRL-2 instrument is estimated by employing a semi-supervised method based on labeled samples comprising 0.3% of the existing HSRL measurement database at the time of the algorithm development. The labeled samples are cases where ancillary information (e.g., in situ measurements, back-trajectory analysis, and visual identification of plumes from the aircraft) has been used to determine the aerosol type. Observations in the remainder of the dataset are then classified by comparison with the labeled samples using the Mahalanobis distance metric (Burton et al., 2012). The HSRL aerosol classification consists of eight types, described by Burton et al. (2012), based on samples of known type observed in airborne field missions in North America since 2006. These are ice, pure dust, dusty mix, maritime, polluted maritime, urban, smoke, and fresh smoke.

### 2.2 NAAPS

Modeled aerosol profiles are based on the Naval Aerosol Analysis Prediction System (NAAPS, http://www.nrlmry.navy.mil/aerosol/, last access: 16 March 2018, Lynch et al., 2016). NAAPS was developed at the Naval Research Laboratory in Monterey, US and is a 3-D aerosol and air pollution model, which originated from a hemispheric sulfate chemistry model developed by Christensen (1997). Dust, sea salt, and biomass-burning smoke have been added to the original model, and are documented in Westphal et al. (2009), Witek et al. (2007), and Reid et al. (2009), respectively. NAAPS runs for this study were conducted in “offline” mode, utilizing meteorological analysis and forecast fields from the 0.3° Navy Global Environmental Model (NAVGEM; Hogan et al., 2014). We apply 550 nm aerosol optical profile information from NAAPS, including *α* and aerosol optical depth (AOD), for our radiative transfer simulations.

Currently, NAAPS produces 6-day forecasts of SO_2_ (gas), anthropogenic and biogenic fine (ABF, combined sulfate, and organic aerosols), dust, biomass burning smoke, and sea salt mass concentration, with 0.3^°^ resolution at 35 levels (surface to 100 hPa). Several versions are available for this model, but three are used in this research: (a) the operational run (OPS) supported by US Navy Fleet Numerical Meteorological and Oceanographic Command, which is used for real-time naval applications of visibility and electromagnetic propagation and features 2-D assimilation of NASA’s Moderate Resolution Imaging Spectroradiometer (MODIS); (b) a 2-D/3-D assimilation version, which combines the MODIS assimilated AOD analysis with a Fernald (1984) based extinction coefficient retrieval for CALIOP data as an assimilation constraint on the vertical model profile (Campbell et al., 2010; Zhang et al., 2010); and (c) a “free” running model, which does not apply any assimilation and is driven solely by model sources and sinks.

### 2.3 Surface reflectance/albedo (*R*)

Direct aerosol radiative effects are reliant on the *R* (surface albedo) of the underlying surface. The accuracy of radiative transfer modeling strongly depends on surface albedo, which in turn is reliant on the surface type (i.e. ocean, land, sea ice etc.; Lyapustin et al., 2011; Bauer and Menon, 2012; Xu et al., 2017). Therefore, albedo is also an important component of the surface boundary condition in a global weather/climate prediction system. We take the opportunity to evaluate the performance of the *R* obtained from different retrievals within the context of our radiative transfer calculations. Three main datasets are used, which are introduced below.

#### 2.3.1 MAIAC

The Multi-Angle Implementation of Atmospheric Correction for MODIS (MAIAC) is an aerosol retrieval algorithm and atmospheric correction of MODIS data over land. The algorithm works globally over all surface types, although aerosols are not currently retrieved over snow. MAIAC products include the following: a cloud mask; water vapor; AODs and Angstrom parameters; surface spectral bidirectional reflectance factor (BRF); instantaneous BRF (iBRF), which is a specific reflectance for a given observation geometry; and albedo for MODIS land bands 1–7, and ocean bands 8–14. The BRF and albedo are derived from the time series of 8-day measurements, and are generated uniformly at 500 m and 1 km resolution in gridded format. For the purposes of this study, we utilized the BRF at 555 nm (MODIS land band 4, Lyapustin et al., 2011).

#### 2.3.2 AirMSPI

The Airborne Multi-angle SpectroPolarimeter Imager (AirMSPI, Diner et al., 2007) is an eight-band (355, 380, 445, 470, 555, 660, 865, and 935 nm) push-broom camera, measuring polarization in the 470, 660, and 865 nm bands, mounted on a gimbal to acquire multiangular observations over a ±67° along-track range. AirMSPI employs a photoelastic modulator-based polarimetric imaging technique to enable accurate measurements of the degree and angle of linear polarization in addition to radiance (Diner et al., 2013). The recently developed aerosol retrieval algorithm (Xu et al., 2017) was applied to a selected SEAC^4^RS set of AirMSPI observations. Contrary to the HSRL and the radiometers (introduced below) during SEAC^4^RS, AirMSPI was flown aboard NASA’s ER-2 high altitude aircraft. This feature limited our ability to conduct the model evaluation experiment to the relatively few cases where both the DC-8 and ER-2 flew in a reasonably collocated formation.

#### 2.3.3 NAVGEM and albedo

The NAVy Global Environmental Model (NAVGEM; Hogan et al., 2014), the US Navy’s operational weather model, combines a semi-Lagrangian/semi-implicit dynamical core and advanced parameterizations of subgrid-scale moist processes, convection, ozone, and radiation. It consists of 61 vertical levels, and a horizontal resolution of 35 km. NAVGEM meteorological fields of pressure, relative humidity, temperature and ozone are used in this research. Additionally, surface information such as surface pressure, temperature, and albedo are used as input in the radiative transfer model. The albedo values used here are based on the climate data of albedo for 24 types of vegetation and bare-soil albedo at three different wavelengths that includes a factor for ground wetness change. Ocean and lake are specified with a climate constant of 0.09, sea ice is 0.60, and snow is set at 0.84. The land surface climate data comes from the US Geological Survey. The vegetation includes seasonal changes while ground wetness changes every time step. Nonetheless, the reflectance values contained herein are based on global distributions of seasonal variation, and have no dependence on solar zenith angle.

### 2.4 Broadband radiometers

A pair of broadband (BBR) Kipp & Zonen CM 22 pyranometers (solar, SW) and a pair of CG 4 pyrgeometers (infrared, IR) were mounted on the top and bottom of the NASA DC-8 aircraft. The solar and IR instruments are modified and calibrated as described by Bucholtz et al. (2010). The radiometers provide flight-level downwelling and upwelling irradiances between 4.5 and 42 μm for the IR and between 0.2 and 3.6 μm in the SW. These irradiances are compulsory for the comparison with the radiative transfer model (RTM) outputs for atmospheric closure purposes. As such, our proposed experiment requires cloud-free skies in order to reconcile values between the two. Otherwise, the in situ measurements would be contaminated to a degree that the RTM could not resolve. Despite the breadth of data collected during SEAC^4^RS, we were limited to a single day of measurements for this study.

### 2.5 Fu Liou Gu (FLG) radiative transfer model

The FLG radiative transfer (RT) model is used in this study to calculate aerosol and molecular heating rates and surface/top-of-atmosphere (TOA) irradiances. The FLG RT scheme (Gu et al., 2011) is a modified and improved version of the Fu-Liou RT model (Fu and Liou, 1992, 1993), which provides new and better parameterizations for aerosol properties to accommodate more realistic radiative effects compared with observations. We utilize the delta-four-stream approximation for solar radiative flux calculations (Liou et al., 1988) and delta-two-and-four-stream approximation for IR radiative flux calculations (Fu et al., 1997) which are implemented in the model. The solar and IR spectra are divided into 6 and 12 bands, respectively, according to the location of absorption bands. In addition to the principal absorbing gases listed, the calculations take absorption by the H_2_O continuum as well as a number of minor absorbers in the solar spectrum into consideration.

### 2.6 Experimental design

HSRL aerosol observations are matched spatiotemporally to the closest NAAPS/NAVGEM analyses profiles. All versions of NAAPS used in this paper contain extinction (*α*) and AOD profiles from the surface to 100 hPa at 22 (now 35) sigma levels of variable vertical resolution (higher resolution in the lower atmosphere). In order to perform comparisons between model and observed fields, the HSRL data are “reduced” to the same model vertical resolution by employing a nearest neighbor classification constrained to model top and bottom.

Besides the aerosol, FLG requires input of atmospheric background fields. *P, T, q*, and O_3_ profiles are obtained from NAVGEM’s previous analysis time to the flight overpass. The case study (19 August 2013), uses profiles from the analyses corresponding to 15:00 UTC and 18:00 UTC, which are closer to the aircraft overpass for that day. There are four different aerosol profiles used as input: one from HSRL (taken as the true) and three that are obtained from the closest NAAPS analysis (which matches NAVGEM’s analysis time). Besides extinction, both the HSRL and NAAPS datasets also contain aerosol speciation profiles. Therefore, each extinction profile is paired to a corresponding speciation profile that is subsequently matched to the FLG internal optical properties as described below. By the same token, each of the NAAPS analyses profiles correspond to a different assimilation version, as described in Sect. 2.2 (NAAPS 3-D, NAAPS OPS, and NAAPS FREE). A control run (NOAER) is set in a similar fashion, but with no aerosol feedback included. Radiative transfer calculations in FLG are performed on each profile from the surface to TOA (0.1 hPa), and we assume there is no significant aerosol loading above the 100 hPa level (aerosol layers above 100 hPa are padded to 0). This is consistent with the current HSRL observations from SEAC^4^RS, which are simultaneously constrained to aircraft height and surface elevation (the top of the HSRL observations is generally obtained within 7–10 km agl).

Although, as will be discussed, smoke and urban aerosols are the two dominant species during this case study, we performed the RT calculations with each of the four particulate aerosols included in NAAPS. At this point it is important to emphasize that the NAAPS and HSRL aerosol type classifications do not necessarily overlap, so we integrate the HSRL aerosols into the four categories that best match the NAAPS speciation: marine (sea salt for NAAPS), urban, smoke (which combines fresh smoke and smoke), and dust (dust, dusty mix, pure dust), while omitting ice (which is not an aerosol, but is considered within the HSRL species). This interpretation is admittedly speculative.

Similarly, assumptions are made to match these merged HSRL/NAAPS species to the FLG optical properties tables, which are based upon the Optical Properties of Aerosol and Clouds (OPAC) software package (Hess et al., 1998) and its 18 different aerosol models. For the purposes of this study, we utilize soot (SOOT), which is used to represent non-hygroscopic absorbing black carbon of 1 g cm^−3^, neglects the chain-like characteristics, and assumes no coagulation of soluble aerosol (Hess et al., 1998). Urban aerosols represent strong pollution in continental/large city areas, for both water soluble and insoluble substances. For dust, we used mineral transported (MITR), which is used to describe desert dust that is transported over long distances with a reduced amount of large particles that are assumed not to enlarge with increasing relative humidity. Given that our observations do not coincide with a significant amount of dust, the choice of this model is considered incidental, as opposed to the compulsory effort necessary to render one or each of the other three OPAC dust models in some combined form suitable for what is a low-order influence on the results. Finally, despite being on land, we naturally still utilize sea salt to relate the incidental amounts of marine aerosol still resolved by NAAPS.

## 3. Results

### 3.1 Description of the smoke event

Although the SEAC^4^RS field study spanned over several weeks, the necessary collocation of the aircraft observations, combined with the requisite cloud-free conditions from which to most accurately apply the broadband radiometer measurements, occurred on 19 August 2013. The comparisons shown in this paper are all based on this date/time, given that this was the one window of opportunity where all of the instruments were synergistically and strategically operating. Additionally, the case matched a high-loading aerosol event that warranted attention.

**Figure 1.**
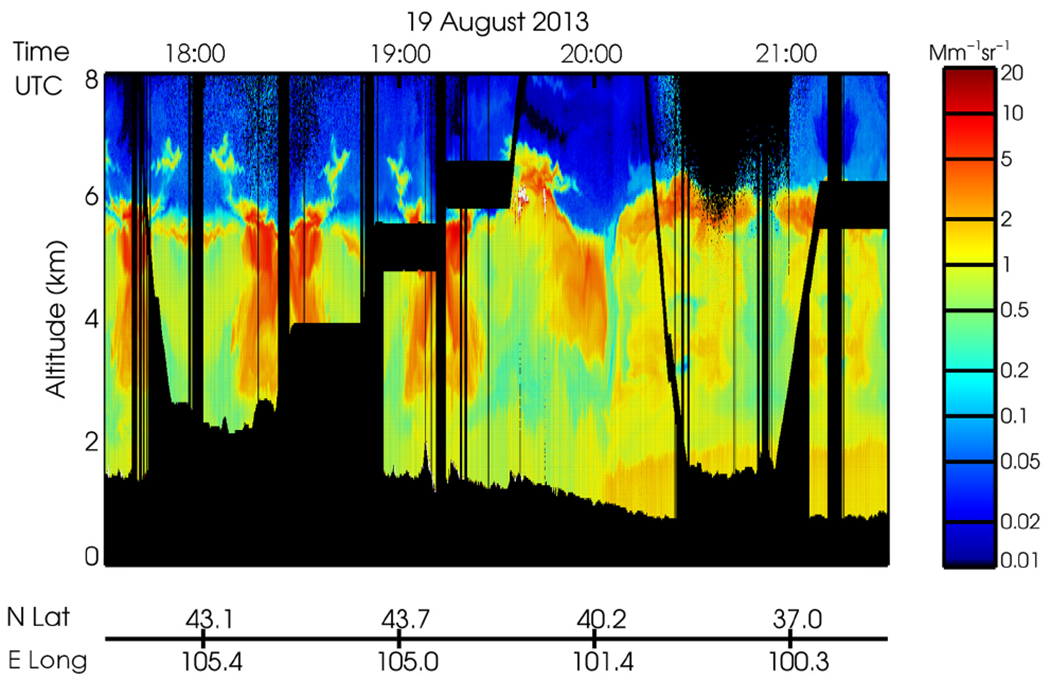
Composite of DIAL/HSRL-2 vertical profiles of the aerosol backscatter coefficient at 532 μm as sampled on the research flight on 19 August 2013.

Figure 1 shows the composite of HSRL-2 vertical profiles of aerosol backscatter coefficient at 532 μm sampled during the flight that day. The enhanced area of laser backscattering near 40° N corresponds with a transported smoke plume that serves as the focus of the study. Fig. 1 depicts a portion of the HSRL flight, which took off from Ellington Field outside of Houston, Texas (29.61° N, 95.16° W; 9.7 m a.m.s.l.), and traveling through the state of Texas and the Thunder Basin Grassland in Wyoming, where the landscape contains intermingled mixed and short-grass prairies in a semiarid climate. This flight sampled the most extensive and thick smoke plume observed during SEAC^4^RS. Within this plume, a profile with an observed peak AOD of 0.73 was sampled at 44.24°N, 104.61°W, at an aircraft cruising altitude of 9.6 km. The plume containing this profile was partially a product of large-scale smoke transport from fire activity in Wyoming, Nebraska, and South Dakota. Back-trajectory analysis for this case (not shown), demonstrates that the air mass originated near the fire regions, less than 24 h before the research flight.

**Figure 2.**
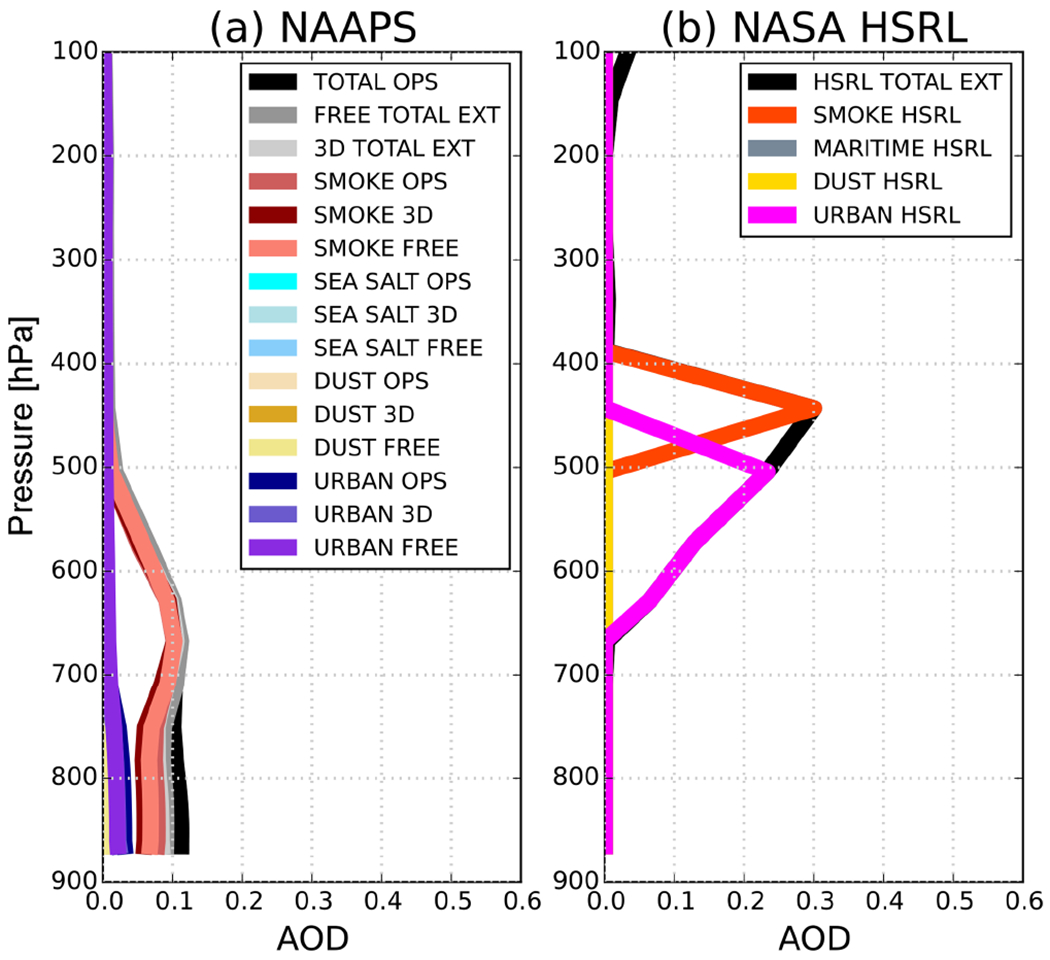
(**a**) NAAPS and (**b**) HSRL aerosol vertical distribution (AODs) for the 19 August case study, with corresponding aerosol speciation.

Figure 2 features the aerosol vertical distribution for the 19 August case study and its corresponding speciation. The profiles depict a 5-minute averaged HSRL segment of the 532 nm aerosol extinction coefficient (km^−1^) centered on 44.24° N, 104.61° W, along with the three corresponding NAAPS 550 nm model profiles from the nearest analysis time. There are significant discrepancies in terms of the aerosol profile structure and the composition when comparing them. The HSRL resolves smoke mostly in the free troposphere, whereas the models constrain the layers to near the surface. Furthermore, AODs between them differ significantly (see Table 1), with the HSRL nearly doubling each of the NAAPS runs.

The HSRL retrieval is dominated by smoke (0.30 AOD) and urban aerosols (0.42 AOD). NAAPS includes four types (smoke, urban, dust, and sea salt as depicted in Table 1). Smoke AODs are within 10%–20% of the observations depending on the run. For the speciated AOD, the largest discrepancy between model and observations is seen in the urban aerosol range, which is significantly misrepresented in all NAAPS runs (−95% in the OPS run, −86% in the 3-D run, and −67% in the FREE run). As suggested above, dust and maritime aerosols (sea salt) are negligible in the HSRL retrievals presented in this publication, and account for only 5% of the total aerosols in the model runs.

The HSRL vs. NAAPS differences are more notable considering the vertical distribution. For all NAAPS runs, the bulk of the aerosol is constrained within 880 and 500 hPa (Fig. 2), while the HSRL discretizes two major aerosol plumes between 400 and 650 hPa associated with urban and smoke aerosols. All model versions failed to properly characterize the aerosol heights and depth of the smoke layer (situated between 400 and 500 hPa). Most notably, the model fails to recognize the presence of a high and elevated urban aerosol layer. As a consequence, besides estimating the radiative budget for this case, we wanted to understand how the differences in vertical distribution between the model and the observations will translate into differences within the radiative forcing field.

### 3.2 Forcing calculations (∇*F*)

**Figure 3.**
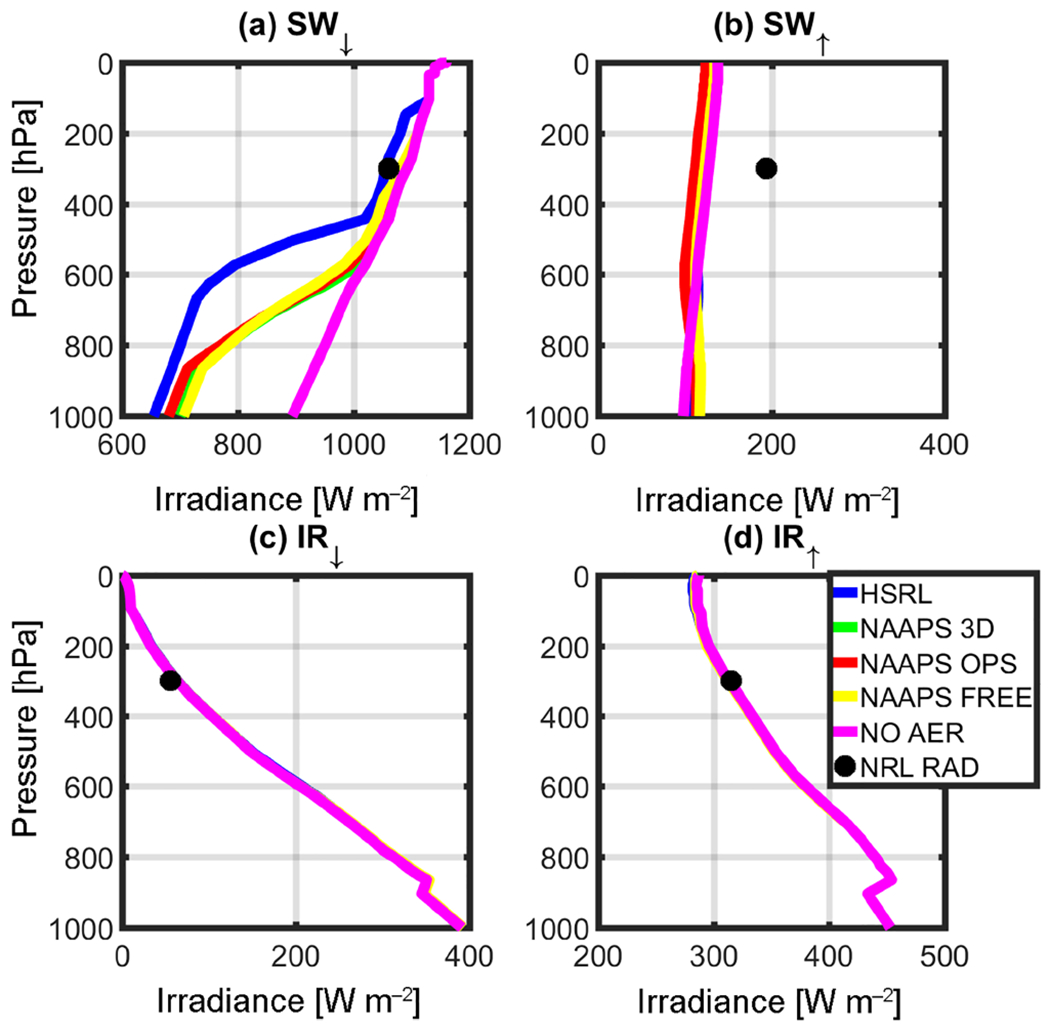
Irradiance calculation results for the (**a**) SW_↓_, (**b**) SW^↑^, (**c**) IR_↓_, and (**d**) IR^↑^ using the MSPI 555 μm reflectance value retrieved for the Thunder Basin case study, 19 August 2013 (BRF=0.166678).

**Figure 4.**
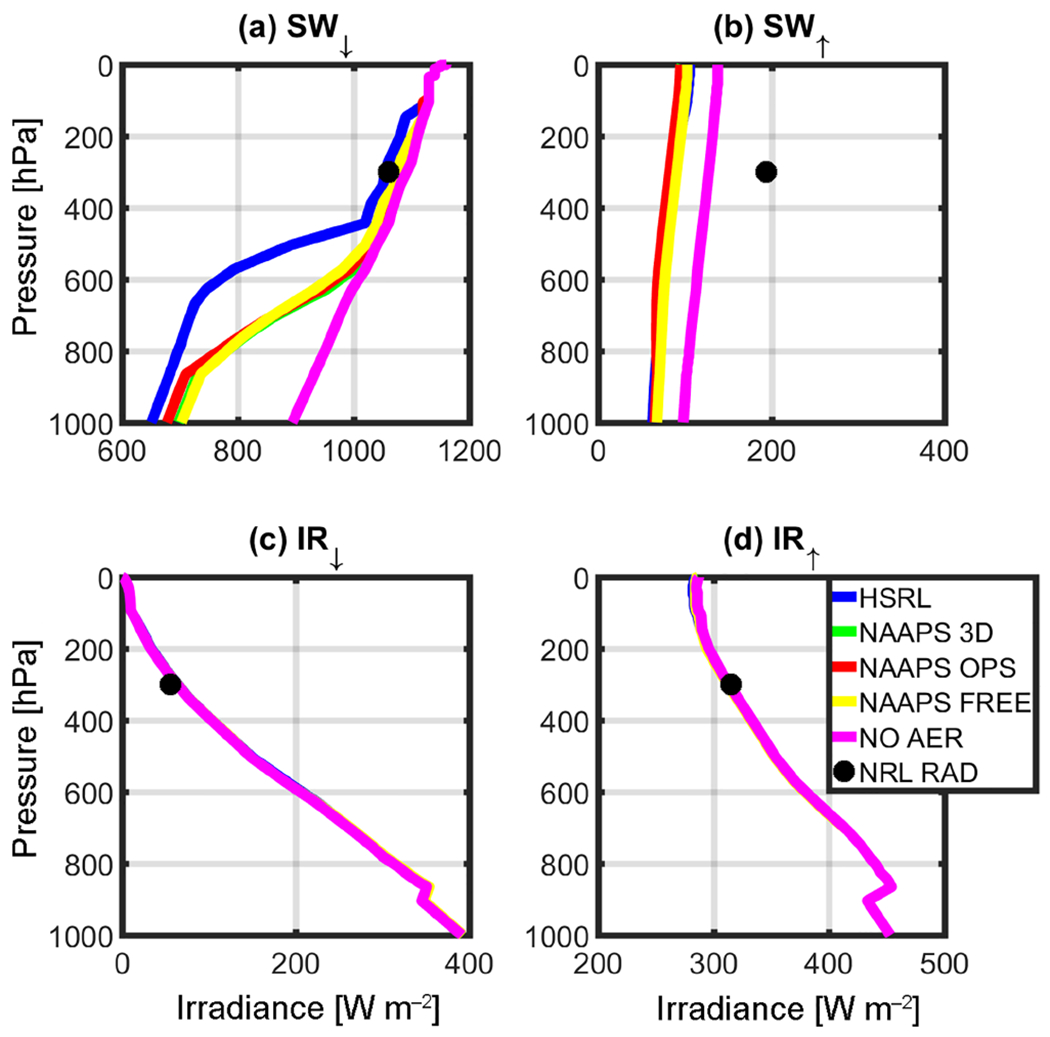
Same as Fig. 3, but with MSPI BB (BRF=0.096711).

**Figure 5.**
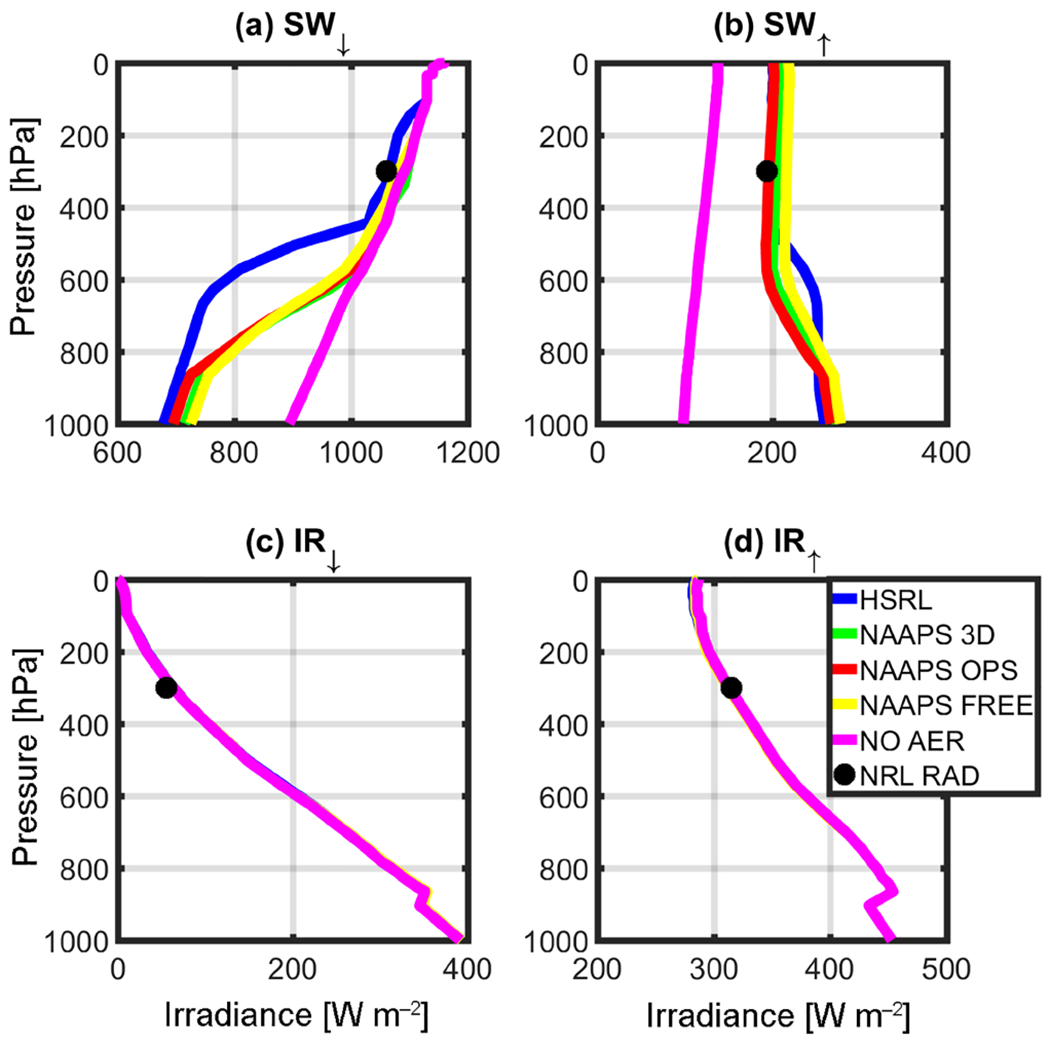
Same as Fig. 3, but with MAIAC 555 μm (BRF=0.5152)

**Figure 6.**
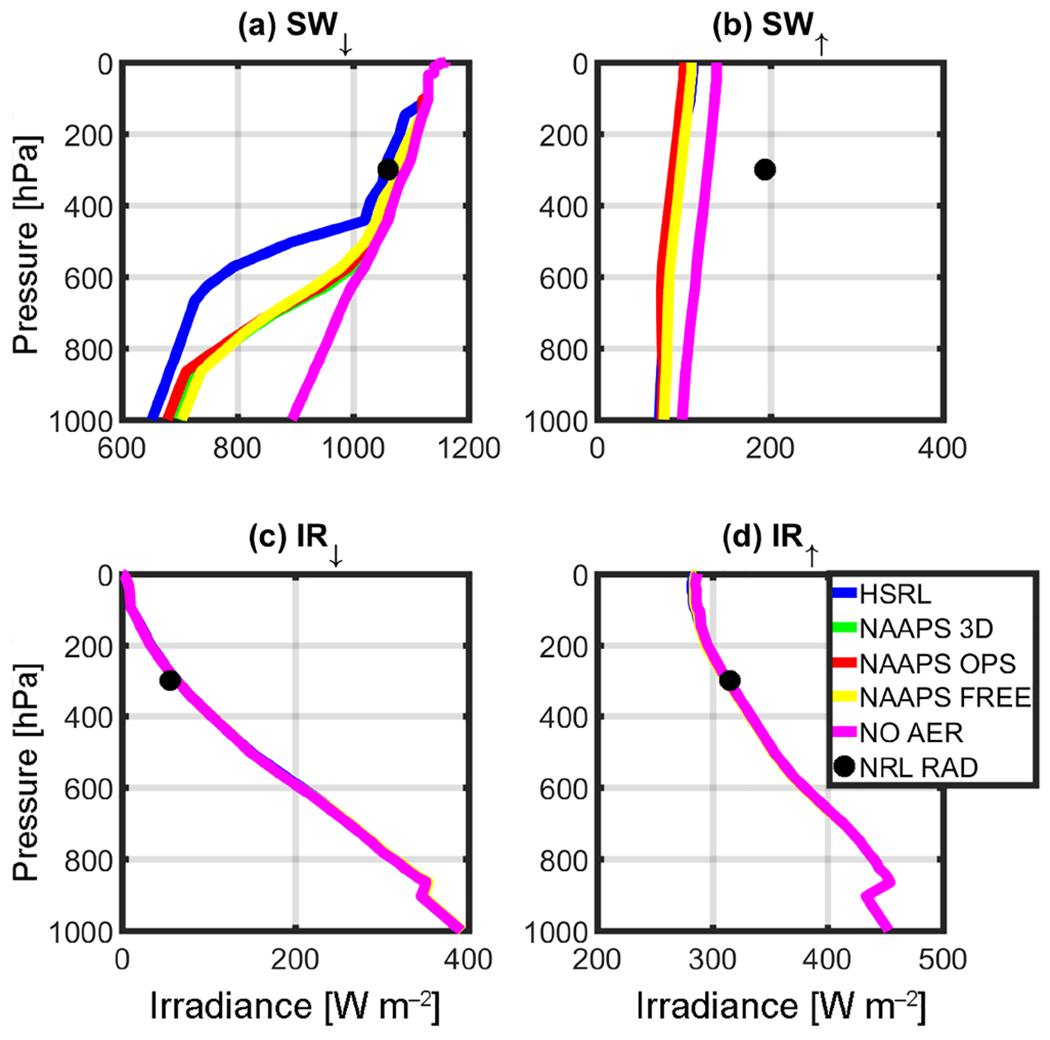
Same as Fig. 3, but with NAVGEM albedo (0.11).

RT simulations were performed after taking the solar zenith angle (SZA) at the corresponding local time and location into consideration, and are depicted in Table 1. Figures 3–6 complement Table 1, showing the results of the instantaneous aerosol radiative forcing for the event. All four figures use the common NAVGEM atmospheric profile as input, with the same climatological ozone profiles but different surface *R* values. Figure 3 considers AirMSPI 555 μm, whereas Fig. 4 is the AirMSPI broadband value for all seven SW channels resolved by FLG. Figure 5 uses the MAIAC 555 μm value, and Fig. 6, uses the NAVGEM climatological albedo corresponding with the closest analysis time to the DC-8 flight. The *R* values used in the four simulations are noted in Table 1.

Output irradiances are contained within the four panels of each figure: (a) downwelling shortwave radiation (SW_↓_), (b) upwelling shortwave radiation (SW^↑^), (c) downwelling infrared radiation (IR^↓^), and (d) upwelling infrared radiation (IR^↑^). Each line corresponds to a different aerosol input (green is NAAPS 3-D, red is NAAPS OPS, yellow is NAAPS FREE, and blue is HSRL-2). The control run (NOAER), which uses NAVGEM *p, T, q*, and *R*, but no aerosol, is shown in magenta. It is noteworthy to mention that the control run does not vary the albedo and is representative of the operational parameters used in NAVGEM (Table 1). The black circle near 300 hPa represents the corresponding observations obtained at the flight level from the airborne broadband radiometers.

The surface reflectance term is of little relevance for the IR calculations. Therefore, there is no associated change in the corresponding IR irradiances across the RT retrievals (Figs. 3–6). Furthermore, due to the relatively small (or non-existent) concentration of larger aerosols (e.g., dust and sea salt, which are active in the IR bands), IR irradiances are primarily driven by the atmospheric state and NAVGEM’s moisture and temperature profiles that initialize FLG. Notably for all cases, IR closure between 1% and 5% was achieved between NAVGEM meteorology and the corresponding simulated irradiances compared with the aircraft measurements. The relatively minor differences in IR forcing are also quantified in Table 2, which summarizes the instantaneous radiative forcing at both the surface and TOA calculated relative to the control run.

The *R* value strongly influences the SW RT estimates. From Figs. 3–6, despite obtaining near-closure in the SW_↓_ term (Figs. 3a, 4a, 5a, 6a), only the outputs with the MAIAC 555 μm BRF (Fig. 5a) approach closure in the SW^↑^. That is, here we compare irradiances with the airborne NRL radiometers mounted on the DC-8. When compared to the radiometers, the HSRL SW^↑^ forcing is within 2% of the airborne radiometer measurements at flight level using the MAIAC *R*. Even with differences in vertical aerosol distribution, the NAAPS model irradiances at flight level are within 10% of the radiometers applying the MAIAC’s reflectances. The values of *R* are undoubtedly far too absorbing in the other simulations when compared to the reference and the radiometer data. Given these results, we focus on the MAIAC’s calculated radiative forcing for the remainder of the discussion.

The reduction in net SW radiation at the surface resulting from smoke/urban aerosols retrieved from the HSRL-2 is −33.00 W m^−2^ when contrasted against the control run (the control run does not include aerosols or clouds). As expected, modeled net SW radiation at the surface is lower due to less aerosol loading (NAAPS OPS = −22.75 W m^−2^, NAAPS 3-D = −10.25 W m^−2^, NAAPS FREE = −4.00 W m^−2^). Magnitude wise, these results compare favorably with Stone et al. (2011), who found a surface direct SW radiative forcing between −65 and −194 W m^−2^ per unit aerosol optical depth during a fire event. Conversely, Toll et al. (2014) found a net impact greater than −100 W m^−2^. Both of these studies examined forcing from very intense fires over more active source regions; however, the AOD values in these previous studies were much higher than the current study (in the order of 1 −4 AOD). At TOA, differences in NOAER and AER irradiances are essentially constrained to the SW^↑^, inducing an overall increase in total irradiances that ranges from +240 (NAAPS FREE) to +256(HSRL) W m^−2^.

Besides understanding the aerosol impact on surface and TOA irradiances, it is important to understand how differences in vertical loading (as depicted in Fig. 2) would impact the vertical distribution of irradiances at the different tropospheric levels. For example, below 900 hPa NAAPS and HSRL irradiances only differ by 8% to 22% in the SW_↓_ and by 10% in the SW^↑^; however, it is noteworthy to mention that the aerosol loading is negligible at those levels in the HSRL retrieval (Fig. 2). Moving upward in the atmospheric column, departures between HSRL and NAAPS irradiances become extremely significant; this is most notable in the middle troposphere (Fig. 5), and particularly in SW_↓_. Figure 5a clearly depicts these differences, with departures between HSRL and model generated irradiances of up to 72%. These differences compare well with the mid-tropospheric smoke/urban aerosol layer in the HSRL profile (Fig. 2). Table 3 summarizes the irradiances (SW^↑^, SW_↓_, and SW_↓TOT_) for this elevated aerosol layer between 500 and 700 hPa for the calculations with the NAAPS profiles, HSRL, and NOAER.

### 3.3 Heating rates

**Figure 7.**
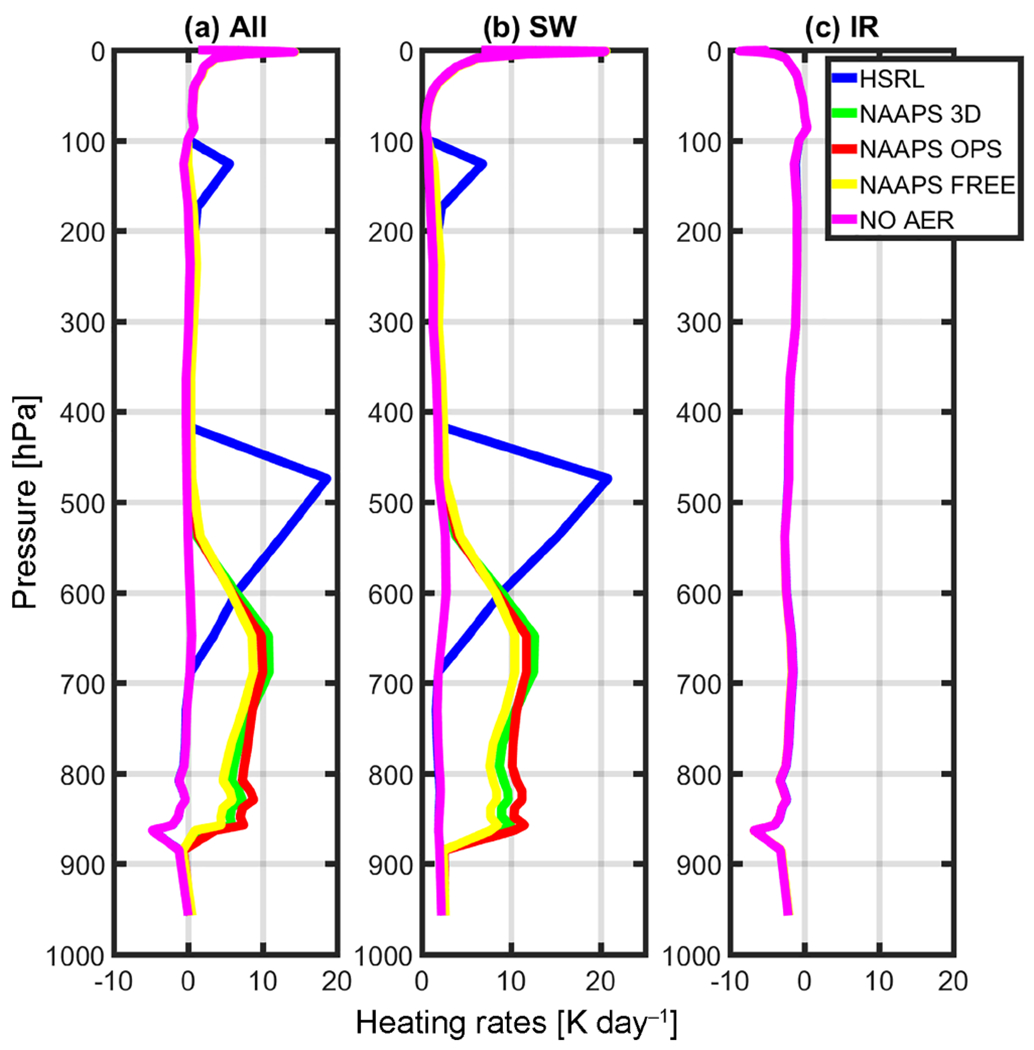
Instantaneous heating rates (IHR) for (**a**) the net, (**b**) SW_Total_, and (**c**) IR using MAIAC 555 μm (BRF=0.5152).

**Figure 8.**
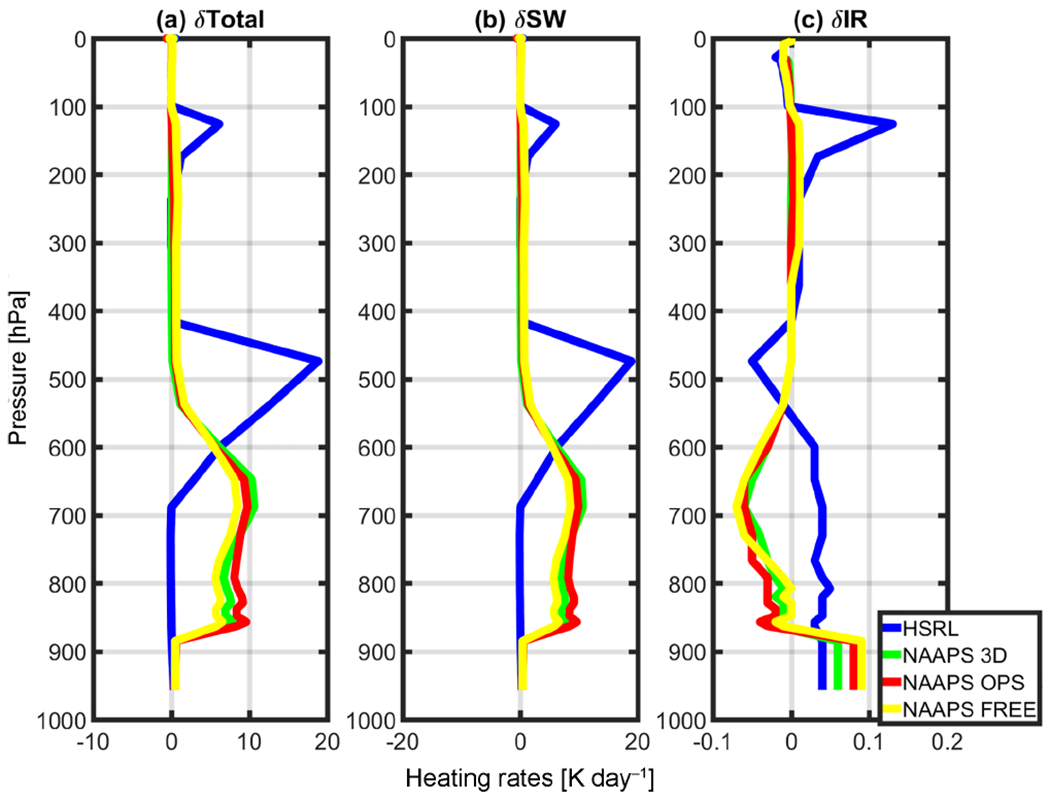
Instantaneous heating rate differences (*δ*(IHR) = NOAER-AER) between all four aerosol profiles (AER=HSRL, NAAPS OPS, NAAPS 3-D, and NAAPS FREE) and the control run (NOAER) using MAIAC 555 μm (BRF=0.5152). The different panels depict (**a**) the net instantaneous heating rates (*δ*IHR_TOT_), (**b**), the SW_Total_ instantaneous heating rates (*δ*IHR_SW_), and (**c**) IR instantaneous heating rates (*δ*IHR_IR_).

Figure 7 depicts the net (total) heating rates using MAIAC 555 BRF, while Fig. 8 shows the relative differences from the NOAER run. Since these observations are not averaged in time (in other words, they are the results of a single observation), they are better referred as “instantaneous” heating rates (IHR). Consistent with the irradiance profiles, IHR profiles similarly correlate with the distributions of each aerosol profile. NAAPS profiles show an increase in net (total) heating with respect to the control run throughout the atmospheric column, although it is more pronounced from 900 to 600 hPa. Heating peaks around 7 K day^−1^ in the lower part of the troposphere.

The HSRL case shows a slight cooling (−0.01 to −0.09 K from 828 to 767 hPa) just below the aerosol layer and a dramatic increase in IHR associated with the aerosol layer found between roughly 700 and 200 hPa (Fig. 8). For this layer, the net heating rates exceed 18 K day^−1^. Recall from Fig. 2, most of the urban/smoke aerosols detected in the HSRL algorithm are located within this layer, which corresponds to over 90% of the HSRL column AOD and relatively high absorptivity at this height. Additionally, the observations were obtained close to the peak of solar noon (10:37 local time, cosine of the SZA = 0.82) during boreal summer. SW IHR is mostly positive at all levels corresponding with detected aerosol. This effect is more noticeable in the HSRL profile due to the higher concentration of soot and urban aerosols.

In contrast to SW, IR heating rates are relatively small and negative (i.e., cooling). As identified above, the HSRL profile does not contain dust or maritime aerosols for this case, which are otherwise highly active in the IR, so we notice a slight warming relative to the control run. Background quantities of sea salt and dust are part of the model runs, and they are significant enough to trigger a slight warming at the surface and a slight cooling within the bulk of the aerosol layer due to the emission of LW radiation. However, this cooling is only in the order of about 0.1 K. There is another area of cooling near the HSRL peak, and a warming of 0.13 K near TOA.

### 3.4 Additional considerations

The RTM results described here are dependent on vertical distribution, total aerosol loading (i.e., AOD), *α, R*, and SZA, for which again due to the limitations of the aircraft experiment we had little clear-sky data to choose from. Thus, we retain the BBR instruments for evaluating column closure. The impact of the vertical distribution has already been addressed within the context of the vertically resolved irradiances and heating rates in the previous two sections. Of significant importance is how the net surface SW irradiances from different NAAPS versions are distinct from each other, even though neither the column AOD nor the aerosol vertical distributions vary dramatically between NAAPS runs. This is primarily due to differences in speciation classification among the profiles, not because of total aerosol loading. In other words, AOD is similar but the speciation distribution is not, so this is a reflection of the radiative forcing efficiency of the aerosol.

Notice in Table 1, that the distribution of urban aerosols is much higher in the NAAPS FREE than in its counterparts, constituting 33% of the total AOD. Urban aerosols only represent 15% of the total AOD in the operational run (NAAPS) and 6% in the NAAPS 3-D. Conversely, smoke is distributed very differently (80% NAAPS, 87% NAAPS 3-D, and 60% NAAPS FREE). FLG utilizes total AOD and the speciation distribution (percentage weights) in the calculations. Therefore, we believe that the differences in the surface (and in the net) SW irradiances are strongly dependent on our choice of aerosol optical properties, which are associated with differences in speciation, and include the single scattering albedo (*ω*_o_) and particle radius. The magnitude of the aerosol forcing is highly sensitive to absorption in the particle size range of anthropogenic aerosols (Nemesure and Schwartz, 1995), which also influences these results. The same can be stated about the HSRL extinction results. Recall that the entire aerosol loading within the HSRL is made up by smoke and urban aerosols, which are concentrated in the same layer. Not only are soot aerosols highly absorbing due to the presence of black carbon, prescribed by the OPAC climatology (i.e., *ω*_o_ of 0.880 at 555 μm, Hess et al., 1998), but OPAC urban aerosols also contain a significant mass density of soot (7.8 mg m^−3^) and high *ω*_o_ (0.817 at 555 *μ*m).

In this study, we do not evaluate sensitivities for any of the optical properties within OPAC via FLG. In reality, this assumption is not necessarily correct, mostly because our speciation does not necessarily match OPAC, but also because the HSRL speciation is recategorized to be similar to NAAPS, as explained in Sect. 2. Therefore, errors in the obtained magnitudes might be associated with this assumption. Additionally, we recognize that the direct radiative effect of absorbing aerosols (smoke/urban) will be different for other cases due to seasonal cycles, time of the day, aerosol loading, and surface characterization. We also recognize that this is an instantaneous result within a portion of a plume, and that the diurnally averaged radiative efficiency for a smoke event might be much lower than for an instantaneous profile alone. However, the key conclusion remains regarding the significance of the vertical representation of aerosols, particularly when calculating irradiances or brightness temperatures throughout the visible and IR spectra.

The modeled aerosol profiles clearly differ from the HSRL observations, in part because the aerosol prognostic model proved unable in this event to resolve aerosol loading and vertical distributions at smaller/regional scales (the model resolution used in this study is (1°)^2^, which is equivalent to 104 km). However, it still brings added value that allows near-closure relative to the observed dataset, something we do not obtain just using the background atmosphere, as we can observe it when contrasting the aerosol forcing and heating rates results with those of the control runs.

### 3.5 The impact on NWP

Aerosol impacts large-scale circulation by virtue of its absorption and vertical distribution. For example, Allen et al. (2012) suggested that the tropical belt expansion may not be driven by stratospheric cooling alone, but may also be impacted by midlatitude heating sources due to aerosol distribution. Additionally, Shen and Ming (2018), examined how aerosol absorption affects the extratropical circulation by analyzing the response to a globally uniform increase in black carbon, and suggested that absorbing aerosols are capable of altering synoptic-scale weather patterns. These studies, among many others, show that these impacts are dependent on the aerosol height, stressing the necessity of better constraining model-simulated aerosol vertical distributions with satellite and field measurements.

One final question for future consideration arising from this work relates to how changes in the vertical distribution of aerosol-induced forcing and heating can potentially impact a forecast cycle, particularly if heating rates of the magnitude exhibited in this case are sustained within one or several data assimilation cycles within the global modeling system. We emphasize this potential in the context of differences in the vertical impact for NAAPS, HSRL, and scale-height aerosol. The distribution of the modeled aerosols (Fig. 2) puts most of the aerosols within 700 hPa, which is a forecast level that is mostly associated with the forecast of precipitation and surface temperatures; in contrast, the scale height distribution of aerosols would put most aerosols within the boundary layer (BL), which is something that would potentially influence near-surface dynamics and diurnal cycles in a model. Conversely, the “aerosol true” (HSRL) peak loading is in the middle of the atmosphere (~500 hPa), which could possibly impact the 1000–500 hPa thickness (influencing temperatures and mid-level jets) and advection fields.

The influence of using near real-time aerosol fields in the data assimilation and NWP fields, and their sensitivity to optical properties, is being studied further; this is not only being undertaken for absorbing aerosols, but for a full aerosol suite and is not constrained to a study region, but globally. Two follow-up publications specifically address these issues, but within the context of dust and sea-salt profiles. Using a 1-D-Var, biases of up to 2 K in temperature and 8 K in dew point were found as a function of optical depth. Additionally, the newly retrieved profiles were substantially improved when compared to aerosol observations. We are also finalizing the inclusion of aerosol-perturbed satellite radiances in the Navy’s data assimilation system, where we have observed significant impacts on the relative humidity and temperature innovations, and an increase of more than 20% in the number of observations that pass quality control for all hyperspectral sensors across the board.

## 4. Conclusions

We have conceptualized the aerosol radiative impact of an inline aerosol analysis field coupled with a global meteorological forecast system by applying the Fu-Liou-Gu four-stream radiative transfer model to data resolved by an offline global aerosol transport model and operational global numerical weather prediction model, utilizing the Navy Aerosol Analysis and Prediction System (NAAPS) and Navy Global Environmental Model (NAVGEM) analysis and surface albedo fields. Model simulations were compared with in situ validation data collected during the NASA 2013 Studies of Emissions and Atmospheric Composition, Clouds and Climate Coupling by Regional Surveys (SEAC^4^RS) experiment, including airborne high spectral resolution lidar (HSRL), an Airborne Multi-angle SpectroPolarimetric Imager (AirMSPI), simultaneous up/down SW and IR irradiance measurements, as well as NASA Moderate Resolution Infrared Spectroradiometer (MODIS) surface reflectance characterization (Multi-Angle Implementation of Atmospheric Correction for MODIS; MAIAC) over Wyoming in the US upper central plains on 19 August 2013. Our goal is a first-order characterization of model fidelities in depicting significant aerosol forcing features in the event that NAAPS and NAVGEM were operated in a coupled configuration, using in situ measurements to demonstrate potential column radiative closure as a verification reference.

The results highlight significant differences between the aerosol loading and vertical distribution between the NAAPS aerosol profiles and those obtained from the HSRL observations in this unique case study. Moreover, we demonstrate the sensitivity that different aerosol distributions exhibit on radiative fluxes and heating rates, specifically in this case associated with solar-absorbing smoke and urban aerosols. Due to the nature of the dominant aerosols in this study, most of this impact is the SW forcing and heating. We observe a reduction of the net SW radiation with the HSRL profile of −33.00 W m^−2^, −22.75 W m^−2^ with the NAAPS operational 2-D-Var assimilation run (OPS), −10.25 W m^−2^ with NAAPS 3-D-Var, and −4.00 W m^−2^ with the free-running aerosol model (NAAPS FREE). We additionally tested the impact that different reflectances/albedos could have on the forcing results, using values from AirMSPI, NAVGEM (i.e., climatology), and MAIAC. Our results demonstrate that the best characterization for this case study was that provided by MAIAC, as it was the only BRF/albedo that allowed us to achieve closure in upward shortwave irradiance, as measured with the BBR array on board the NASA DC-8.

Instantaneous heating rates for the NAAPS model runs peaked around 7Kday^−1^ in the lower part of the troposphere, while the HSRL profiles resulted in values of up to 18 K day^−1^ in the middle of the troposphere. The magnitudes and vertical placement of such peaks are directly proportional to the magnitude of the aerosol loading and distribution. Furthermore, there are limitations imposed by the model resolution. Horizontally, the model is very coarse (104 km, global domain) when compared with a single point observation. Vertically, the model resolution is higher in the lower troposphere and coarser in the middle of the atmosphere; therefore, this points to another possible reason why the model misses the mid-tropospheric smoke enhancement. Additionally, we acknowledge there are other factors influencing these magnitudes including solar zenith angle, selected optical properties, and surface characterization, and that these results are subject to seasonality.

We highlight two additional closing points to this study. First, this was a relatively simple experiment, achievable within the broad data collection effort that SEAC^4^RS represented. However, in order to apply the airborne radiometers as a direct closure proxy for comparing the radiative transfer simulations cloud-free skies were a necessity, which severely limited how much of the SEAC^4^RS archive we could evaluate. Nevertheless, the community needs to recognize the value in the simplicity of this effort, either through coordinated airborne study and a Lagrangian view or combined surface radiation measurements paired with high-resolution, multi-spectral lidar measurements like a HSRL that directly constrain aerosol optical properties. The pending revolution of coupled aerosol/global meteorological models will prove ripe motivation for the aerosol community with respect to developing such studies and providing the vigorous verification and sensitivity analysis embraced by operational meteorological modeling groups.

However, this point raises another obvious need with respect to the diversity of aerosol scenes and the impact on such evaluation. That is, in this case we consider smoke and urban aerosol, which are reasonably well constrained within the OPAC database (leaving aside for the moment the evolution of smoke in transport events, and thus how well OPAC really captures such optical properties). Dust, however, is seemingly a far more complicated consideration. OPAC contains four different dust models, and their infrared impact is something that was not a primary consideration with smoke and urban aerosols in this case. Consequently, this study likely represents a relatively simple case; thus, it is again necessary that the community invests in closure studies aimed at aerosol diversity, and particularly dust, in order to thoroughly understand inline performance and sensitivities.

## Figures and Tables

**Table 1.** Parameters utilized to initialize the FLG radiative model. Values correspond to the Thunder Basin case study on 19 August 2013. (SZA represents solar zenith angle).

Date	19 August 2013				
	
Coordinates	44.24° N 104.61° W				
	
cos^−1^(SZA)	0.82				
	
Surface temperature	301.070 K				
	
Surface pressure	1010.16 hPa				
	
MSPI 555 BRF	0.166678				
	
MSPI BB BRF	0.096711				
	
MAIAC 555 BRF	0.5152				
	
NAVGEM Albedo	0.11000				

Aerosol	Total AOD	Smoke	Urban	Maritime	Dust

NAAPS	0.40	0.32	0.06	0.01	0.01
NAAPS 3-D	0.33	0.29	0.02	0.01	0.01
NAAPS	0.42	0.25	0.14	0.01	0.02
FREE					

HSRL-2	0.73	0.30	0.43	0.00	0.00

**Table 2.** Instantaneous radiative forcing results at the surface and top-of-atmosphere (TOA) for the Thunder Basin case study, utilizing the MAIAC 555 nm reflectances.

	SFC IRRADIANCE (W m^−2^)				TOA IRRADIANCE (W m^−2^)			
	
	HSRL	NAAPS OPS	NAAPS 3-D	NAAPS FREE	HSRL	NAAPS OPS	NAAPS 3-D	NAAPS FREE
SW^↑^	−215.00	−208.25	−200.75	−197.00	−259.00	−258.25	−248.50	−241.00
SW_↓_	−248.00	−231.00	−211.00	−201.00	0.00	0.00	0.00	0.00
Total SW	−33.00	−22.75	−10.25	−4.00	259.00	258.25	248.50	241.00
IR^↑^	0.00	0.00	0.00	0.00	−3.00	−1.00	0.00	−1.00
IR_↓_	0.00	1.00	0.00	1.00	0.00	0.00	0.00	0.00
Total IR	0.00	1.00	0.00	1.00	−3.00	−1.00	0.00	−1.00
Total (SW + IR)	−33.00	−21.75	−10.25	−3.00	256.00	257.00	248.50	240.00

**Table 3.** Instantaneous SW radiative forcing (in W m^−2^) calculated values, corresponding to HSRL smoke/urban aerosol layers in the middle of the troposphere (see Fig. 2b).

	*δ*SW^↑^ (W m^−2^)					*δ*SW_↓_ (W m^−2^)					*δ*SW_TOT_ (W m^−2^)				
	
Pressure (hPa)	HSRL	3-D	OPS	FREE	NOAER	HSRL	3-D	OPS	FREE	NOAER	HSRL	3-D	OPS	FREE	NOAER
504	215	201	193	214	461	904	1040	1030	1020	1060	688	839	837	806	599
572	236	200	194	215	461	809	1010	999	989	1040	573	810	806	774	579
628	248	207	199	221	461	764	961	951	943	1020	517	754	752	722	559
667	251	216	206	228	461	746	915	907	904	1010	495	699	701	675	549
708	252	226	215	237	461	737	868	863	866	997	485	642	648	628	536

## References

[R1] AirMSPI L1 data: available at: https://eosweb.larc.nasa.gov/project/airmspi, last access: 6 May 2018.

[R2] Allen RJ, Sherwood SC, Norris JR, and Zender CS: The equilibrium response to idealized thermal forcings in a comprehensive GCM: implications for recent tropical expansion, Atmos. Chem. Phys, 12, 4795–4816, 10.5194/acp-12-4795-2012, 2012.

[R3] Alpert P, Krichak SO, Tsidulko M, Shafir H, and Joseph JH: A dust prediction system with TOMS initialization, Mon. Weather Rev, 130, 2335–2345, 10.1175/1520-0493(2002)130&lt;2335:ADPSWT&gt;2.0.CO;2, 2002.

[R4] Bauer SE and Menon S: Aerosol direct, indirect, semidirect, and surface albedo effects from sector contributions based on the IPCC AR5 emissions for preindustrial and present-day conditions, J. Geophys. Res, 117, D01206, 10.1029/2011JD016816, 2012.

[R5] Bucholtz A, Hlavka DL, McGill MJ, Schmidt KS, Pilewskie P, Davis SM, Reid EA, and Walker AL: Directly measured heating rates of a tropical subvisible cirrus cloud, J. Geophys. Res, 115, D00J09, 10.1029/2009JD013128, 2010.

[R6] Burton SP, Ferrare RA, Hostetler CA, Hair JW, Rogers RR, Obland MD, Butler CF, Cook AL, Harper DB, and Froyd KD: Aerosol classification using airborne High Spectral Resolution Lidar measurements – methodology and examples, Atmos. Meas. Tech, 5, 73–98, 10.5194/amt-5-73-2012, 2012.

[R7] Burton SP, Ferrare RA, Vaughan MA, Omar AH, Rogers RR, Hostetler CA, and Hair JW: Aerosol classification from airborne HSRL and comparisons with the CALIPSO vertical feature mask, Atmos. Meas. Tech, 6, 1397–1412, 10.5194/amt-6-1397-2013, 2013.

[R8] Campbell JR, Reid JS, Westphal DL, Zhang J, Hyer EJ, and Welton EJ: CALIOP aerosol subset processing for global aerosol transport model data assimilation, IEEE J. Sel. Top. Appl, 3, 203–214, 10.1109/jstars.2010.2044868, 2010.

[R9] Carmona I, Kaufman Y, and Alpert P: Using numerical weather prediction errors to estimate aerosol heating, Tellus B, 60, 729–741, 2008.

[R10] Christensen JH: The Danish eulerian hemispheric model-A three dimensional air pollution model used for the arctic, Atmos. Environ, 24, 4169–4191, 1997.

[R11] Diner DJ, Davis A, Hancock B, Gutt G, Chipman RA, and Cairns B: Dual photoelastic modulator-based polarimetric imaging concept for aerosol remote sensing, Appl. Optics, 46, 8428–8445, 2007. 10.1364/ao.46.008428 18071373

[R12] Diner DJ, Xu F, Garay MJ, Martonchik JV, Rheingans BE, Geier S, Davis A, Hancock BR, Jovanovic VM, Bull MA, Capraro K, Chipman RA, and McClain SC: The Airborne Multiangle SpectroPolarimetric Imager (AirMSPI): a new tool for aerosol and cloud remote sensing, Atmos. Meas. Tech, 6, 2007–2025, 10.5194/amt-6-2007-2013, 2013.

[R13] Fernald FG: Analysis of atmospheric lidar observations: Some comments, Appl. Opt, 23, 652–653, 10.1364/AO.23.000652, 1984. 18204618

[R14] Fu Q and Liou KN: On the correlated k-distribution method for radiative transfer in nonhomogeneous atmospheres, J. Atmos. Sci, 49, 2139–2156, 1992.

[R15] Fu Q and Liou KN: Parameterization of the radiative properties of cirrus clouds, J. Atmos. Sci, 50, 2008–2025, 1993.

[R16] Fu Q, Liou KN, Cribb MC, Charlock TP, and Grossman A: Multiple scattering parameterization in thermal infrared radiative transfer, J. Atmos. Sci, 54, 2799–2812, 1997.

[R17] Gu Y, Liou KN, Ou SC, and Fovell R: Cirrus cloud simulations using WRF with improved radiation parameterization and increased vertical resolution, J. Geophys. Res, 116, D06119, 10.1029/2010JD014574, 2011.

[R18] Hair JW, Hostetler CA, Cook AL, Harper DB, Ferrare RA, Mack TL, Welch W, Izquierdo LR, and Hovis FE: Airborne high spectral resolution lidar for profiling aerosol optical properties, Appl. Optics, 47, 6734–6752, 10.1364/AO.47.006734, 2008. 19104525

[R19] Haywood JM, Allan RP, Culverwell I, Slingo T, Milton S, Edwards J, and Clerbaux N: Can desert dust explain the outgoing longwave radiation anomaly over the Sahara during July 2003?, J. Geophys. Res, 102, 6831–6864, 2005.

[R20] Hess M, Koepke P, and Schult I: Optical properties of aerosols and clouds: the software package OPAC, B. Am. Meteorol. Soc, 79, 831–844, 1998.

[R21] Hogan TI, Liu M, Ridout JS, Peng MS, Whitcomb TR, Ruston BC, Reynolds CA, Eckermann SD, Moskaitis JR, Baker NL, McCormack JP, Viner KC, McLay JG, Flatau MK, Xu L, Chen C, and Chang SW: The Navy Global Environmental Model. Special Issue on Navy Operational Models, Oceanography, 27, 10.5670/oceanog.2014.73, 2014.

[R22] IPCC: Climate Change 2014: Synthesis Report Contribution of Working Groups I, II and III to the Fifth Assessment Report of the Intergovernmental Panel on Climate Change, Core Writing Team, edited by: Pachauri RK and Meyer LA, IPCC, Geneva, Switzerland, 151 pp., 2014.

[R23] Jacobson MZ and Kaufman YZ: Wind reduction by aerosol particles, Geophys. Res. Lett 33, L24814, 10.1029/2006GL027838, 2006.

[R24] Liou KN, Fu Q, and Ackerman TP: A simple formulation of the delta-four-stream approximation for radiative transfer parameterizations, J. Atmos. Sci, 45, 1940–1947, 1988.

[R25] Lyapustin A, Martonchik J, Wang Y, Laszlo I, and Korkin S: Multiangle implementation of atmospheric correction (MAIAC): 1. Radiative transfer basis and look-up tables, J. Geophys. Res, 116, D03210, 10.1029/2010JD014985, 2011.

[R26] Lynch P, Reid JS, Westphal DL, Zhang J, Hogan TF, Hyer EJ, Curtis CA, Hegg DA, Shi Y, Campbell JR, Rubin JI, Sessions WR, Turk FJ, and Walker AL: An 11-year global gridded aerosol optical thickness reanalysis (v1.0) for atmospheric and climate sciences, Geosci. Model Dev, 9, 1489–1522, 10.5194/gmd-9-1489-2016, 2016.

[R27] Milton SF, Greed G, Brooks ME, Haywood J, Johnson B, Allan RP, and Grey WMF: Modelled and observed atmospheric radiation balance during West Africa dry season: Role of mineral dust, biomass burning aerosol, and surface albedo, J. Geophys. Res, 113, D00C02, 10.1029/2007JD009741, 2008.

[R28] Mulcahy JP, Brooks ME, and Milton SF: Aerosol impacts in the Met Office global NWP model, paper presented at the 2010 EGU General Assembly, Vienna, Austria, 2–7 May 2010.

[R29] Mulcahy JP, Walters DN, Bellouin N, and Milton SF: Impacts of increasing the aerosol complexity in the Met Office global numerical weather prediction model, Atmos. Chem. Phys, 14, 4749–4778, 10.5194/acp-14-4749-2014, 2014.

[R30] Ramanathan V, Crutzen PJ, Kiehl JT, and Rosenfeld D: Aerosol, climate and the hydrological cycle, Science, 294, 2119–2124, 2001. 11739947 10.1126/science.1064034

[R31] Randles CA, DaSilva AM, Buchard V, Colarco PR, Darmenov A, Govindarajua R, Smirnov A, Holben B, Ferrare R, Hair J, Shinozuka Y, and Flynn CJ: The MERRA-2 aerosol reanalysis, 1980 Onward. Part I: System description and data assimilation evaluation, J. Climate, 30, 6823–6850, 10.1175/JCLI-D-16-0609.1, 2017. PMC5859955 29576684

[R32] Reid JS, Hyer EJ, Prins EM, Westphal DL, Zhang J, Wang J, Christopher SA, Curtis CA, Schmidt CC, Eleuterio DP, Richardson KA, and Hoffman JP: Global Monitoring and Forecasting of Biomass-Burning Smoke: Description of and Lessons from the Fire Locating and Modeling of Burning Emissions (FLAMBE) Program, IEEE J. Sel. Top. Appl, 2, 144–162, 2009.

[R33] Rodwell MJ and Jung T: Understanding the local and global impacts of model physics changes: an aerosol example, Q. J. Roy. Meteor. Soc, 134, 1479–1497, 2008.

[R34] SEAC^4^RS: HSRL data: available at: https://www-air.larc.nasa.gov/missions/seac4rs/index.html, last access: 6 May 2018.

[R35] Shen Z and Ming Y: The Influence of Aerosol Absorption on the Extratropical Circulation, J. Climate, 31, 5961–5975, 10.1175/JCLI-D-17-0839.1, 2018.

[R36] Stone RS, Augustine JA, Dutton EG, O’Neill NT, and Saha A: Empirical determinations of the longwave and shortwave radiative forcing efficiencies of wildfire smoke, J. Geophys. Res, 116, D12207, 10.1029/2010JD015471, 2011.

[R37] Tegen I, Hollrig P, Chin M, Fung I, Jacob D, and Penner J: Contribution of different aerosol species to the global aerosol extinction optical thickness: estimates from model results, J. Geophys. Res.-Atmos, 102, 23895–23915, 1997.

[R38] Toll V, Gleeson E, Nielsen KP, Mannik A, Masek J, Rontu L, and Post P: Impacts of the direct radiative effect ofaerosols in numerical weather prediction over Europe using the ALADIN-HIRLAM NWP system, Atmos. Res, 172-173, 163–173, 10.1016/j.atmosres.2016.01.003, 2016.

[R39] Toll V, Gleeson E, Nielsen KP, Mannik A, Masek J, Rontu L, and Post P: Impacts of the direct radiative effect of aerosols in numerical weather prediction over Europe using the ALADIN-HIRLAM NWP system, Atmos. Res, 172-173, 163–173, 10.1016/j.atmosres.2016.01.003, 2016.

[R40] Tompkins AM, Cardinali C, Morcrette JJ, and Rodwell M: Influence of aerosol climatology on forecasts of the African easterly jet, Geophys. Res. Lett, 32, L10801, 10.1029/2004GL022189, 2005.

[R41] Toon OB, Maring H, Dibb J, Ferrare R, Jacob DJ, Jensen EJ, Luo ZJ, Mace GG, Pan LL, Pfister L, Rosenlof KH, Redeman J, Reid JS, Singh HB, Thompson AM, Yokelson R, Minnis P, Chen G, Jucks KW, and Pszenny A.: Planning, implementation, and scientific goals of the Studies of Emissions and Atmospheric Composition, Clouds and Climate Coupling by Regional Surveys (SEAC4RS) field mission, J. Geophys. Res.-Atmos, 121, 4967–5009, 10.1002/2015JD024297, 2016.

[R42] Westphal DL, Curtis CA, Liu M, and Walker AL: Operational aerosol and dust storm forecasting, WMO/GEO Expert Meeting on an International Sandand Dust Storm Warning System, IOP Conf. Series: Earth and Environmental Science, 7, 10.1088/1755-1307/7/1/012007, 2009.

[R43] Witek ML, Flatau PJ, Quinn PK, and Westphal DL: Global sea-salt modeling: results and validation against multicampaign shipboard measurements, J. Geophys. Res, 112, D08215, 10.1029/2006JD007779, 2007.

[R44] Xu F, van Harten G, Diner DJ, Kalashnikova OV, Seidel FC, Bruegge CJ, and Dubovik O: Coupled retrieval of aerosol properties and land surface reflection using the Airborne Multiangle SpectroPolarimetric Imager, J. Geophys. Res.-Atmos, 122, 7004–7026, 10.1002/2017JD026776, 2017.

[R45] Zhang J and Reid JS: A decadal regional and global trend analysis of the aerosol optical depth using a data-assimilation grade over-water MODIS and Level 2 MISR aerosol products, Atmos. Chem. Phys, 10, 10949–10963, 10.5194/acp-10-10949-2010, 2010.

[R46] Zhang J, Campbell JR, Reid JS, Westphal DL, Baker NL, Campbell WF, and Hyer EJ: Evaluating the impact of assimilating CALIOP-derived aerosol extinction profiles on a global mass transport model, Geophys. Res. Lett, 38, L14801, 10.1029/2011GL047737, 2011.

[R47] Zhang J, Campbell JR, Hyer EJ, Reid JS, Westphal DL, and Johnson R: Evaluating the impact of multi-sensor data assimilation on a global aerosol particle transport model, J. Geophys. Res.-Atmos, 119, 4674–4689, 10.1002/2013JD020975, 2014.

[R48] Zhang J, Reid JS, Christensen M, and Benedetti A: An evaluation of the impact of aerosol particles on weather forecasts from a biomass burning aerosol event over the Midwestern United States: observational-based analysis of surface temperature, Atmos. Chem. Phys, 16, 6475–6494, 10.5194/acp-16-6475-2016, 2016.

